# Insight into Temperature Dependence of GTPase Activity in Human Guanylate Binding Protein-1

**DOI:** 10.1371/journal.pone.0040487

**Published:** 2012-07-11

**Authors:** Anjana Rani, Esha Pandita, Safikur Rahman, Shashank Deep, Apurba Kumar Sau

**Affiliations:** 1 National Institute of Immunology, Aruna Asaf Ali Marg, New Delhi, India; 2 Department of Chemistry, Indian Institute of Technology, New Delhi, India; University of Oldenburg, Germany

## Abstract

Interferon-γ induced human guanylate binding protein-1(hGBP1) belongs to a family of dynamin related large GTPases. Unlike all other GTPases, hGBP1 hydrolyzes GTP to a mixture of GDP and GMP with GMP being the major product at 37°C but GDP became significant when the hydrolysis reaction was carried out at 15°C. The hydrolysis reaction in hGBP1 is believed to involve with a number of catalytic steps. To investigate the effect of temperature in the product formation and on the different catalytic complexes of hGBP1, we carried out temperature dependent GTPase assays, mutational analysis, chemical and thermal denaturation studies. The Arrhenius plot for both GDP and GMP interestingly showed nonlinear behaviour, suggesting that the product formation from the GTP-bound enzyme complex is associated with at least more than one step. The negative activation energy for GDP formation and GTPase assay with external GDP together indicate that GDP formation occurs through the reversible dissociation of GDP-bound enzyme dimer to monomer, which further reversibly dissociates to give the product. Denaturation studies of different catalytic complexes show that unlike other complexes the free energy of GDP-bound hGBP1 decreases significantly at lower temperature. GDP formation is found to be dependent on the free energy of the GDP-bound enzyme complex. The decrease in the free energy of this complex at low temperature compared to at high is the reason for higher GDP formation at low temperature. Thermal denaturation studies also suggest that the difference in the free energy of the GTP-bound enzyme dimer compared to its monomer plays a crucial role in the product formation; higher stability favours GMP but lower favours GDP. Thus, this study provides the first thermodynamic insight into the effect of temperature in the product formation of hGBP1.

## Introduction

Human guanylate binding protein-1 (hGBP1) belongs to a family of large farnesylated GTPases that is induced by interferon-γ, an immunomodulatory cytokine [1]–[3]. It is a member of the Dynamin superfamily with molecular mass of about 65–70 kDa [4]. Unlike other GTPases, this protein has the ability to hydrolyze GTP to a mixture of GDP and GMP with GMP being the major product of the reaction at physiological temperature, 37°C [5]. The crystal structure of hGBP1 has been solved in the absence and presence of the β-γ resistant substrate analogue, GppNHp [6], [7]. The protein is 592 residues long consisting of mainly two domains, a large globular αβ-domain [1–278] and a C-terminal elongated purely α-helical domain (312–592) [6], [7]. These two domains are joined by a short intermediate region (279–311) [7]. The protein exists as a monomer and dimer in the absence and presence of the analogue GppNHp, respectively [6]. This protein has been shown to inhibit VSV (vesicular stomatitis virus) and EMCV (Encephalomyocarditis virus) in HeLa cells [8], [9]. It also controls the endothelial cell proliferation by inhibiting the matrix metalloproteinase-1 [10], [11].

**Figure 1:**
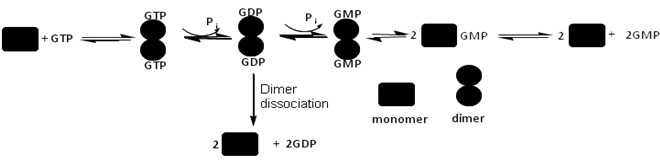
Scheme shows the substrate induced dimerization of hGBP1 and hydrolysis of GTP. After the first phosphate cleavage, the GDP-bound enzyme dimer can undergo the second phosphate cleavage leading to the formation of GMP or irreversibly dissociates to give free GDP.

 Like other large GTPases, hGBP1 has been shown to undergo nucleotide concentrations dependent oligomerization and stimulation of the GTPase activity [6]. The biochemical and structural studies of this protein have been done extensively. The hydrolysis of GTP occurs through successive cleavages of the phosphates, since PP_i_ was not obtained as a reaction product [5]. The role of the individual domains in the hydrolysis of GTP has been investigated [12]. The N-terminal globular domain (1–278), similar in structure to the small GTPases like Ras, exhibits only the first hydrolysis i.e. GDP formation [12]. But the C-terminal helical domain has been shown to stimulate the GTPase activity [12]. We reported that the intermediate region plays a critical role in GMP formation through allosteric interaction and thus acts as an internal GAP [12]. We also recently reported that the intermediate region is important for dimerization and regulation of GTPase activity in hGBP1 [13]. The hydrolysis of GTP by hGBP1 is shown in Figure 1, where the mechanism is believed to involve with a number of catalytic steps before the products get released from the active site of the enzyme. It has been suggested that after the first phosphate cleavage, GDP-bound enzyme dimer irreversibly dissociates to give GDP as a minor product of the reaction [14], [15]. It is also reported that GDP became significant when the hydrolysis of GTP by hGBP1 was carried out at 15°C [5], suggesting that the formation of GDP and the subsequent phosphate cleavage are strongly regulated by an unknown mechanism. This also indicates that the hydrolysis of GTP in this protein is affected strongly with temperature. At temperature lower than the physiological GDP is more prevalent, while at the physiological temperature, GMP formation becomes more favourable. Although, there is not much information about how this enzyme actually provides resistance against the viruses, but several reports show that various microbial infections can alter body’s temperature [16], [17]. Since hGBP1 shows differential product formation with temperature, it is possible that this protein follows an altered defence mechanism under different stress conditions. This has prompted us to investigate a detailed understanding of how the hydrolysis of GTP in hGBP1 is controlled by temperature. In the present study, we addressed the following key issues in hGBP1; i) how does temperature control the GTPase activity in hGBP1? ii) unlike GMP, why GDP formation is unusually different with temperature (GDP is lower and higher at 37 and 15°C, respectively) and iii) whether the stability of the nucleotide bound dimeric protein has a role in GMP formation? The temperature dependent kinetic assays using radiolabeled (α-^32^P) GTP on hGBP1 as well as assays with external GDP reveal that GDP formation takes place as a result of the reversible dissociation of GDP-bound enzyme dimer to GDP-bound enzyme monomer that subsequently dissociates into the free enzyme and GDP. The study also demonstrates that unlike the free protein and other catalytic complexes the thermodynamic stability of GDP-bound hGBP1 complex decreases significantly with temperature and the lower stability of this complex at low temperature (10°C) compared to at high (37°C) is the reason for higher GDP formation at low temperature. The data also suggest that the stability of the nucleotide bound dimer compared to its nucleotide free monomer is important for GMP formation. The temperature dependent study has allowed us to construct an energy diagram for GDP formation from the GDP-bound enzyme dimer.

## Materials and Methods

### Mutagenesis

 The mutant hGBP1 proteins, Ser157Ala and Glu313Ala were generated using QuikChange Site-Directed Mutagenesis Kit (Stratagene, USA) and pProExHTa-hGBP1 as a template, according to the manufacturer’s protocol. The positive mutants were then identified by DNA sequencing.

### Protein Expression and Purification

Wild type and mutant proteins of hGBP1 were over-expressed and purified as described earlier [12]. The concentration of the proteins was determined using a Bio-Rad protein dye assay. The concentrations of the proteins were measured in triplicate and the final concentration was determined based on the average.

### Assay for GTPase Activity

 GTPase assays were carried out in a buffer containing 50 mM Tris-HCl pH 8.0, 100 mM KCl, 5 mM MgCl_2_, 0.2 mM DTT, and 6.6 nM radiolabeled (α-^32^P)GTP (3000 Ci/mmol, Perkin Elmer) depending on the nature of the experiment. The concentration of unlabeled GTP [Sigma] was used as indicated.

The steady-state kinetic experiments for the wild-type and mutant proteins were performed by incubating the enzyme with a fixed amount of radiolabeled (α-^32^P) GTP and varying concentrations of the unlabeled GTP (micromolar to millimolar) at different temperature, as reported earlier [12]. The reaction was stopped by adding 0.166 M EDTA (final concentration). The nucleotides were separated by thin-layer chromatography on polyethyleneimine cellulose sheet using 0.75 M KH_2_PO_4_, pH 3.5, as a developing solvent. The intensities of the bands were quantified using a FLA-5100 phosphorimager (Fuji, Japan).

For all experiments, initial rates for the formations of GDP and GMP were determined. The experimental data were fitted to a Hill equation to obtain apparent Michaelis constant (*K_m_*), maximum velocity (*V_max_*), and Hill coefficient (n).

### Gel Filtration Assays

The analytical gel filtration chromatography was performed using a Perkin Elmer HPLC system, USA. A Phenomenex S 4000 column was used. The mobile phase contained 50 mM Tris pH 8.0, 100 mM KCl and 5 mM MgCl_2_. 200 µM GppNHp was also added in the mobile phase wherever the experiment was carried out in the presence of the substrate analogue. A flow rate of 1 ml/min was used. A standard curve was generated from the elution volumes of proteins with known molecular weight such as amylase (200 kDa), alcohol dehydrogenase (150 kDa), bovine serum albumin (66 kDa), carbonic anhydrase (29 kDa) and cytochrome c (12.4 kDa). The molecular weight of the protein was determined based on the retention volume.

### Fluorescence Measurements

The fluorescence measurements were carried out on spectrofluorimeters from Varian (Model # Cary, Eclipse) as well as Fluoromax-4 (Horiba Scientific). The fluorescence excitation wavelength of 295 nm was used to eliminate the contribution from amino acids other than tryptophan. The emission spectra were recorded from 310 to 400 nm. The monochromator slit width was kept at 5 nm for excitation and 5 nm for emission measurements respectively, unless stated otherwise. All measurements were done using 20 mM Tris-HCl, 5 mM MgCl_2_, 100 mM KCl, pH 7.5 at room temperature. The fluorescence measurements of the protein samples were carried out with an optical density of below 0.1 at 295 nm to avoid inner filter effect. However, for samples with optical density higher than 0.1 at 295 nm, inner filter correction was done using the equation, F_corr_ = F_obs_* Antilog (OD_ex_ + OD_em_)/2, where F_obs_ and F_corr_ are the observed and corrected fluorescence respectively. OD_ex_ and OD_em_ are the optical densities at the excitation and emission wavelengths respectively.

 Heat-induced denaturation studies were carried out using a spectrofluorimeter. The temperature of the sample was raised from 20°C to 85°C using a peltier thermostat, coupled with the spectrofluorimeter. The rate of sample heating was about 1°C/min and the data was recorded at the interval of 0.2°C. The excitation wavelength was 295 nm and the fluorescence intensity was measured at the emission maxima. To check the reversibility, the protein was thermally denatured (temperature was used higher than the *T*_m_) and cooled down to room temperature, and the sample was reheated. Similar thermal profile and *T*_m_ were observed upon reheating, suggesting that unfolding occurs in a reversible manner.

### Determination of Thermodynamic Parameters

#### A. Urea-induced denaturation studies

Urea-induced transition curves of hGBP1 were monitored by following a change in tryptophan fluorescence at pH 7.5 and 37°C. Using a non-linear least-square curve fitting method, the denaturant-induced transition was analyzed for Δ*G*_D_ and *m*_g_ following the relation [18]–[19],
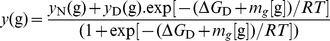
(1)where, *y* (g) is the observed optical properties at [g], g is the molar concentration of urea, *y*_N_ and *y*_D_ are optical properties of the native and denatured protein molecules under the same experimental conditions in which *y* (g) was measured, Δ*G*_D_ is the value of Gibbs energy change in the absence of denaturant, *m*_g_ is the slope (∂Δ*G*_D_/∂[g])_T,P_, *R* is the universal gas constant and *T* is the temperature in Kelvin. The concentration of urea was determined by measuring the refractive index. Analysis of each urea-induced transition curve was done using a two-state model and [g]-dependencies of *y*_N_(g) and *y*_D_(g) are linear (i.e., *y*_N_(g) = *a*_N_ + *b*_N_ [g] and *y*_D_(g) = *a*_D_ + *b*_D_ [g], where *a* and *b* are [g]-independent parameters, and subscripts N and D represent for the native and denatured protein molecules, respectively).

#### B. Heat-induced denaturation studies

Heat-induced denaturation studies of hGBP1 were carried out by monitoring the fluorescence of tryptophan. The thermodynamic parameters like *T*_m_ (the mid-point of the thermal denaturation) and Δ*H*_m_ (the enthalpy change at *T*_m_) were extracted from the thermal profile by a non-linear least square analysis using Eq. 2 assuming a two-state model [20]–[22],
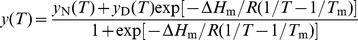
(2) where *y*(*T*) is the optical property at temperature *T* (K), *y*_N_(*T*) and *y*_D_(*T*) are the optical properties of the native and denatured states of the protein at temperature *T*(K) and *R* is the molar gas constant. In the analysis of each heat-induced transition curve, it was assumed that a parabolic function describes the dependence of the optical properties of the native and denatured protein molecules (i.e., *y*_N_(*T*) = *a*_N_ + *b*_N_ *T* + *c*_N_ *T* ^2^ and *y*_D_(*T*) = *a*_D_ + *b*_D_ *T* + *c*_D_ *T* ^2^, where *a*_N_, *b*_N_, *c*_N_, *a*_D_, *b*_D_, and *c*_D_ are temperature-independent coefficients) [21]. The temperature-independent specific heat capacity at constant pressure (Δ*C*_p_) was determined from the slope of the linear plot of Δ*H*_m_ versus *T*_m_, using the following relation:



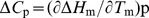



Using values of *T*_m_, Δ*H*_m_ and Δ*C*_p_, the value of Δ*G*_D_ at any temperature *T*, Δ*G*_D_(*T*) was estimated with the help of the Gibbs-Heltmoltz equation:
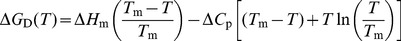
(3)


## Results and Discussion

### Temperature Dependent Studies Reveal Existence of Reversible Intermediates during the Dissociation of GDP-bound Enzyme Dimer

**Figure 2:**
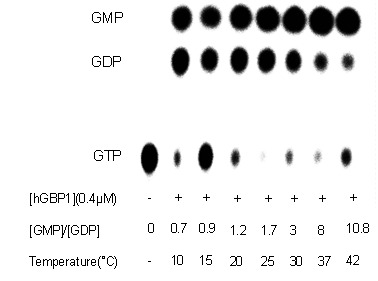
Temperature dependent GTPase assays of wild type hGBP1. The experiments were carried out by mixing a trace amount of radiolabeled (α-^32^P) GTP and 50 μM of unlabelled GTP.

**Figure 3:**
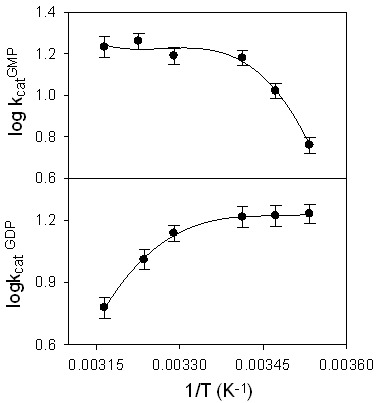
Logarithmic plot of *k*_cat_ for GMP and GDP formation versus 1/T shows the nonlinear trend. The temperature dependent GTPase assays were carried out as described in the material and methods. The solid lines drawn through the experimental data points show the trend of the plot.

Unlike at 37°C, GDP became significant when the hydrolysis of GTP was carried out by the wild type protein at 15°C [5]. This suggests that GDP formation increased with decrease in temperature. As suggested in Figure 1, after the first phosphate cleavage, GDP formation occurs through an irreversible dissociation of GDP-bound enzyme dimer. To understand how temperature regulates the product formation in hGBP1 and whether GDP formation takes place through irreversible dissociation of GDP-bound enzyme dimer, we carried out temperature dependent GTPase assays using radiolabeled (α-^32^P) GTP, as shown in Fig. 2. Analysis of the data shows that unlike GMP, GDP decreases with increase in the temperature (Fig. 2). As a result, the ratio of GMP to GDP is ∼1 at lower temperature (10–20°C) but increases significantly at higher temperature (25–42°C). To get further insight into temperature dependence of GDP formation, steady-state kinetics of the GTPase assay using radiolabeled (α-^32^P) GTP by the wild type protein was carried out in the temperature range of 10–42°C. The steady-state kinetic assays were carried out similar to the described earlier [12]. The data were fitted to a Hill equation to obtain the kinetic parameters *K*_cat_ (rate constant for the product formation, called catalytic turnover) and *K*_m_ (apparent affinity for the substrate) at each temperature and the Arrhenius plots were made for each product. The Arrhenius plot for GMP formation (logarithmic plot of *k*_cat_ versus 1/T) shows a nonlinear curve (Fig. 3). In practice, the Arrhenius plot for one step reaction shows a linear behaviour. The nonlinear dependence of *k*_cat_ for GMP with temperature (Fig. 3) clearly indicates that after the substrate binding, the conversion of GTP-bound enzyme complex to GMP must be associated with at least more than one step. This implies that *k*_cat_ for GMP is controlled by more than one rate constant, which is consistent with Fig. 1, where the GTP-bound enzyme dimer undergoes a number of catalytic steps before GMP is released from the active site. This type of nonlinear Arrhenius plot was observed in xanthine oxidase, where more than one step was found during the breakdown of the enzyme-substrate complex to the product [23]. More interestingly, the Arrhenius plot for GDP formation shows a nonlinear curve with positive slope in the temperature range of 30–42°C (Fig. 3). This indicates that GDP formation decreases with increase in the temperature and the activation energy at this temperature range is associated with a negative value. The activation energy of a reaction step cannot be negative and is not physically meaningful. Thus, it suggests that the dissociation of GDP from GDP-bound enzyme dimer during the course of reaction is likely to be the result of more than one elementary step, corresponding to a combination of the rate constants. Hence, GDP formation cannot simply take place through one step irreversible dissociation of GDP-bound enzyme dimer, as suggested earlier [14] and is shown in Fig. 1. The first step is likely to be involved with the dissociation of GDP-bound enzyme dimer to GDP-bound enzyme monomer, whereas the second step represents the dissociation of GDP-bound enzyme monomer to free GDP and the enzyme. These arguments suggest that there should be an intermediate during the conversion of GDP-bound enzyme dimer to free GDP and the enzyme, which is shown as below.
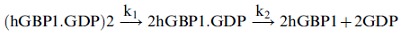
(4)


**Figure 4:**
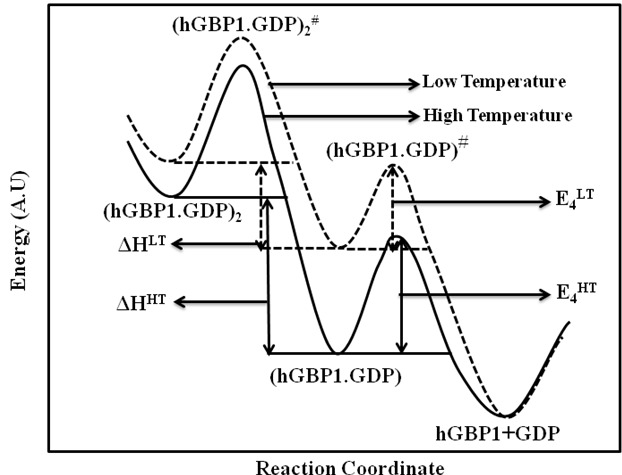
Energy diagram of GDP formation from GDP-bound enzyme dimer. Schematic representation of the energy diagram of (hGBP1.GDP)_2_ dimer to (hGBP1.GDP) monomer and finally free GDP and hGBP1 during GDP formation from GDP-bound enzyme dimer. At high and low temperature, the activation energies were shown to be -ve and zero, respectively. For clarity, other parts of the reaction are not shown.

The above scheme leads to the following equation, *k*_cat_^GDP^ (*k*_cat_ for GDP formation) = k_1_.k_2_/(k_1_+ k_2_), where k_1_ and k_2_ represent the rate constants for the dissociation of GDP-bound enzyme dimer to GDP-bound enzyme monomer and GDP-bound enzyme monomer to GDP, respectively. According to the above equation, the Arrhenius plot will give a straight line with negative slope, if one of the rate constants is negligible with respect to the other. However, the Arrhenius plot for GDP formation shows a straight line with positive slope (i.e. -ve activation energy) within the temperature range of 30–42°C. Hence, the mechanism described in Eq. 4 does not provide a complete description of the reaction. Alternatively, if the mechanism is considered as
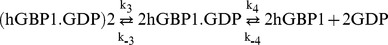
(5)
*k*_cat_^GDP^ can be related to the above rate constants as follows, *k*_cat_^GDP^ = k_3_.k_4_/(k_3_+ k_−3_+ k_4_), where k_3_, k_−3_ and k_4_ represent the rate constants corresponding to Eq. 5 [24]. The above equation would give a linear Arrhenius plot with positive slope, if k_−3_ is much larger than the sum of k_3_ and k_4_. Under this condition, the equation will become, *k*_cat_^GDP^ ∼ (k_3_/k_−3_).k_4_ ∼ K×k_4_ (where K is the equilibrium constant for the first step of Eq. 5). The activation energy in this case would be equal to the sum of the enthalpy change for the first step and the activation energy for the second. Thus, the observed activation energy in the temperature range of 30–42°C could be negative, if the first step becomes an exothermic reaction and the heat released associated with this step (ΔH^HT^) is more than the activation energy of the next step (E_4_^HT^, see Fig. 4). This possibility is in good agreement with our experimental data. Therefore, the mechanism suggested in Eq. 5 appears to be valid. Additionally, it suggests that there is reversibility between GDP-bound enzyme dimer and GDP-bound enzyme monomer at higher temperature (30–42°C). The GDP-bound enzyme monomer further reversibly dissociates into the free enzyme and GDP. However, these individual rate constants cannot be determined from the present set of experimental data.

As observed in Fig. 3, at low temperature (10–20°C) the Arrhenius plot for GDP formation is linear with zero slope. This suggests that at lower temperature the activation energy associated with GDP is zero. This is possible, if the first step of the reaction described in Eq. 5 becomes exothermic and the heat change associated with this step (ΔH^LT^) is equal to the activation energy for the next step (E_4_^LT^, see Fig. 4). This is in good agreement with our experimental data and therefore suggests that the decrease in temperature will favour the dissociation of GDP-bound enzyme dimer to GDP-bound enzyme monomer. This also clearly indicates that GDP formation from GDP-bound enzyme dimer is tightly regulated with temperature.

**Figure 5:**
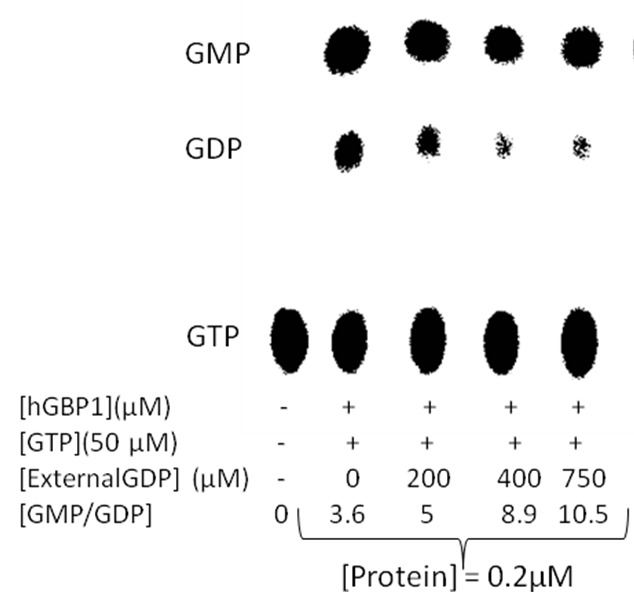
GTPase assays of wild type hGBP1 at various concentrations of external GDP at 37 °C. The concentration of unlabelled GTP was kept approximately 35 fold higher than the Kd of GppNHp for wild type hGBP1 (Kd for GppNHp to whGBP1 ~ 1.5 μM).

**Figure 6:**
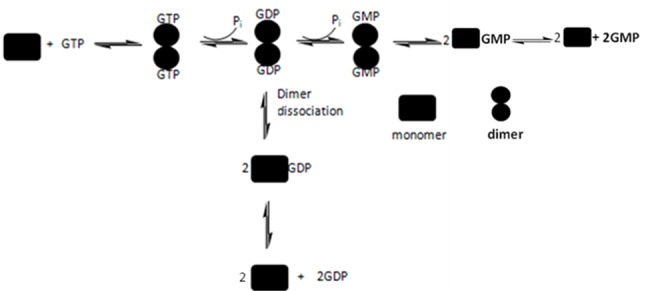
Substrate induced dimerization of hGBP1 and the hydrolysis of GTP. After the first phosphate cleavage, the GDP-bound enzyme dimer undergoes the second phosphate cleavage leading to the formation of GMP or reversibly dissociates into GDP-bound enzyme monomer, which further reversibly dissociates into free GDP and enzyme.

To verify the reversibility at higher temperature, we carried out GTPase assays of wild type hGBP1 using radiolabeled (α-^32^P) GTP in the presence of varying concentrations of external GDP as shown in Fig. 5. GDP is not an inhibitor of hGBP1 catalyzed hydrolysis reaction of GTP [5]. Analysis of the data showed that GMP formation was marginally affected with increasing concentrations of external GDP. However, GDP formation was significantly reduced (Fig. 5). As a result, the ratio of GMP to GDP increases with increasing concentrations of external GDP. This suggests that the presence of external GDP reduces the dissociation of GDP-bound enzyme monomer to free GDP and the enzyme resulting in an increase in the ratio of GMP to GDP. This clearly indicates the reversibility between GDP-bound enzyme monomer, and free GDP and the enzyme (last step of Eq. 5). Thus, it validates our kinetic analysis and strongly support that GDP-bound enzyme dimer reversibly dissociates into GDP-bound enzyme monomer, which ultimately gives GDP as a product through reversible dissociation of GDP-bound enzyme monomer as shown in Fig. 6. This also agreed well with GMP as a predominant product at higher temperature, where GDP-bound enzyme dimer preferably undergoes subsequent phosphate cleavage yielding GMP-bound enzyme dimer, which ultimately produces GMP.

### Thermodynamic Insight into the difference in Product Formation at Different Temperature

**Figure 7:**
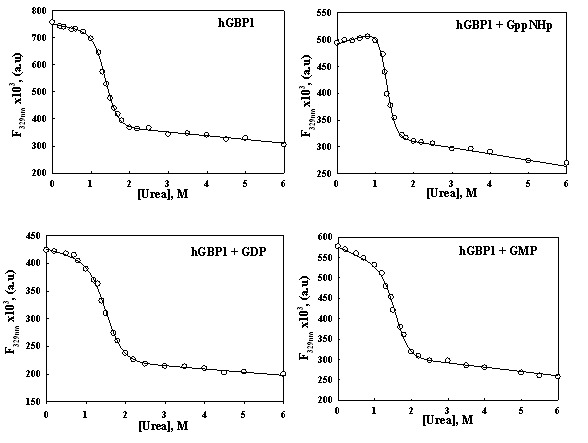
Urea- induced denaturation studies of wild type hGBP1 in the absence or presence of various nucleotides as indicated in each panel. The concentrations of the protein, GppNHp, GDP and GMP were kept 0.5, 100, 500 and 500 μM respectively. The mixture of wild type protein and the nucleotide was prepared in a reaction buffer containing 20 mM Tris-HCl, pH 7.5, 100 mM KCl and 5 mM MgCl_2_ and kept for an hour at 37 °C. Then the reaction mixture was incubated with urea at 37 °C. The fluorescence intensity at the emission maxima was plotted against concentration of urea. The solid lines drawn through the experimental data points show the fitted curves using Eq 1. The experiments were carried in triplicate.

To get thermodynamic insight into the temperature dependence of the product formation (ratio of GMP to GDP) in hGBP1, we have carried out urea-induced unfolding studies to determine the stability of wild type protein in the presence of various nucleotides so that they may represent the catalytic complexes that are formed during the course of GTP hydrolysis, as shown in Fig. 1. This was carried out by measuring the intrinsic tryptophan fluorescence of wild type hGBP1 alone or in the presence of the β-γ resistant GTP-analogue GppNHp, GDP and GMP, separately with increasing concentrations of urea at 37°C, since the wild type protein contains four tryptophans in the globular domain. Wild type hGBP1 exists as a monomer in the absence or presence of either GDP or GMP but becomes a dimer with GppNHp [7]. The fluorescence intensity at the emission maxima of wild type protein was plotted against the concentrations of urea and the experimental data was fitted using Eq. 1 to obtain the Δ*G*_D_° (Fig. 7). Similar urea- induced denaturation studies of wild type protein were carried out in the presence of GppNHp, GDP or GMP (Fig. 7). The data were analyzed similar to wild type alone to obtain the Δ*G*_D_ (Table 1). The values of Δ*G*_D_ at 37°C for the free, GppNHp-bound, GDP-bound and GMP-bound proteins are 5.4, 7.1, 4.3 and 5.2 kcal/mol, respectively. This implies that the binding of the nucleotides alters the stability of the protein. The data also suggests that the stability of the protein in the presence of GppNHp increases compared to the free protein, which is consistent with our earlier result [12], where the nucleotide binding increases the molar ellipticity of the wild type protein. Interestingly, the GDP-bound protein is found to be less stable than the free protein, but the GMP-bound complex shows similar stability to the free protein. In order to compare the data with biochemical assays, similar urea-induced denaturation studies were carried out to determine the Δ*G*_D_ at 10°C for the free protein as well as all catalytic complexes. However, we could not determine the reliable value of Δ*G*_D_ at 10°C because of inconsistent data. This could be due to the solubility of urea at low temperature.

**Table 1:** Thermodynamic data extracted from the urea-induced denaturation studies, as described in materials and methods.

Protein	Urea-induced unfolding
	Δ*G*_D_ (kcal.mol^−1^) at 37°C
**hGBP1**	5.4±0.5
**hGBP1+GppNHp**	7.1±0.4
**hGBP1+GDP**	4.3±0.3
**hGBP1+GMP**	5.2±0.3

Urea-induced denaturation study was carried out with varying concentrations of urea fitted to Eq. 1 to obtain Δ*G*_D_.

**Figure 8:**
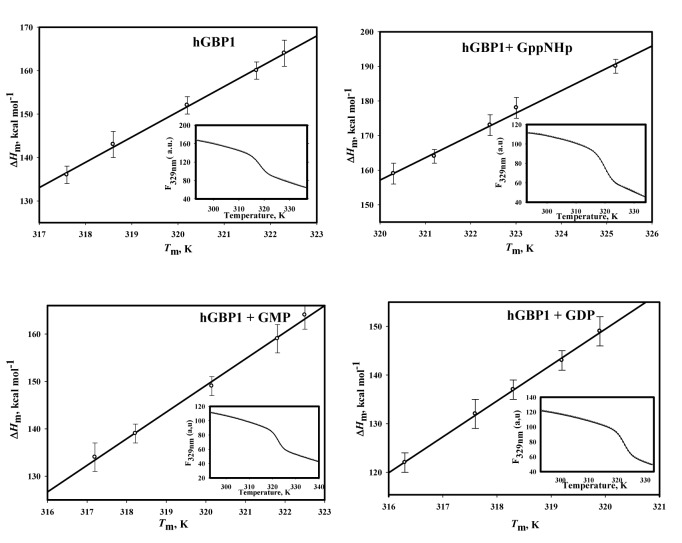
▵*H*_m_ versus *T*_m_ plot for wild type hGBP1 in the absence or presence of various nucleotides. Plots of *▵H*_m_ vs *T*_m_ are shown for wild type hGBP1 in the absence or presence of GppNHp, GDP and GMP. The concentration of free protein and nucleotides were kept identical to urea-induced denaturation study. Heat-induced denaturation studies of the protein were carried out by measuring tryptophan fluorescence intensity from 20 to 85 °C with varying concentrations of urea (0-0.8 M). The data were fitted to Eq. 2 to obtain ▵*H*_m_ and *T*m at each concentration of urea. The slope of the solid line drawn through the data points was used for the estimation of ▵*C*_p_. The inset represents heat-induced denaturation curve in the absence of urea and the solid line drawn through the data points shows the fitted curve according to Eq. 2. The experiments were carried in triplicate.

To determine the Δ*G*_D_ at low temperature, we carried out experiments in an alternative way following the reported procedure [20]–[22]. This requires estimation of the following thermodynamic parameters; Δ*C*_p_ (specific heat capacity of denaturation), *T*_m_ (melting temperature) and Δ*H*_m_ (enthalpy change at *T*_m_). To determine these, we carried out heat-induced unfolding studies of wild type protein by measuring the tryptophan fluorescence with varying concentrations of urea (Fig. 8, inset). The data were fitted using Eq. 2 to obtain Δ*H*_m_ and *T*_m_. *T*_m_ decreased with an increase in the concentration of urea from 49.3±0.4°C in the absence of urea to 44.6±0.2°C in the presence of 0.8 M urea. Similarly, Δ*H*_m_ was decreased with increasing concentrations of urea. Δ*H*_m_ values were plotted against *T*_m_ (Fig. 8) and the slope of the straight line of the Δ*H*_m_ versus *T*_m_ plot was used to evaluate Δ*C_p_*. It was determined to be 5.9 kcal.mol^−1^.K^−1^ for the free protein. Similar thermal unfolding measurements of wild type protein in the presence of GppNHp, GDP or GMP, separately were carried out with varying concentrations of urea (Fig. 8 inset). The plot of Δ*H*_m_ vs *T*_m_ showed straight lines for all complexes (Fig. 8) and the ΔC_p_ values were evaluated similar to the free protein (Table 2). Using the thermodynamic parameters shown in Table 2 and Eq. 3, the Δ*G*_D_ at 10°C for these complexes were determined. The values of Δ*G*_D_ at 10°C for the free, GppNHp-bound, GDP-bound and GMP-bound proteins are 5.2, 5.9, 0.5 and 5.6 kcal/mol, respectively (Table 2). We also determined the Δ*G_D_* of these complexes at 37°C in this way to compare the data with the chemical denaturation studies. The values of Δ*G*_D_ at 37°C for the free, GppNHp-bound, GDP-bound and GMP-bound proteins are 4.8, 6.5, 3.4 and 5.0 kcal mol^−1^, respectively. The stability at 37°C obtained by two different methods showed a good correlation, which validates our analysis. The stability of the free and GppNHp-bound proteins at 10°C is similar to at 37°C. But for GMP-bound protein it is slightly more stable at 10°C compared to at 37°C. In contrast, GDP-bound hGBP1 exhibits significant lower stability at 10°C compared to at 37°C (0.5 versus 3.4 kcal/mole at 10 versus 37°C respectively). Interestingly, at 37°C the stability of the protein in the presence of GppNHp increases compared to the free protein suggesting that the substrate binding increases in the stability of the protein. But the Δ*G*_D_ of the GDP-bound hGBP1 is found to be lower than the GppNHp-bound hGBP1 at both these temperatures. This suggests that after the first hydrolysis GDP-bound protein becomes less stable compared to GppNHp-bound protein as well as the free protein at both temperatures. On the other hand, the stability of the protein in the presence of GMP is almost similar to the free protein at both temperatures indicating that the binding of GMP does not alter the stability of the protein. After the first hydrolysis, the difference in the stability of GDP-bound hGBP1 complex at these two temperatures determines whether the formation of GDP could be higher or lower. At 37°C, after the first hydrolysis the GDP-bound hGBP1 complex undergoes preferably the second hydrolysis leading to the formation of GMP-bound hGBP1 complex. But at 10°C the GDP-bound enzyme complex will preferably dissociate leading to GDP formation rather than the second hydrolysis. The data show that after the first hydrolysis as well as release of the first phosphate, the stability of the GDP-bound protein complex plays a crucial role in the product formation. This finding agreed very well why GDP is a minor product at 37°C but became significant at 10°C. Since wild type protein does not dimerize with either external GDP or GMP, the stability of the dimeric protein with these nucleotides cannot be determined.

**Table 2:**
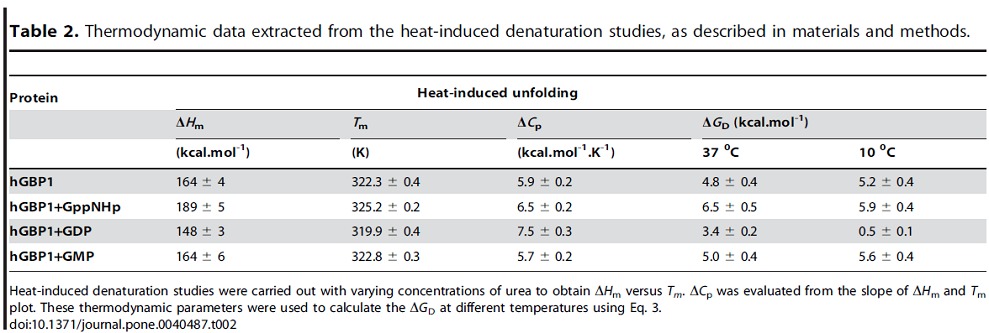
Thermodynamic data extracted from the heat-induced denaturation studies, as described in materials and methods.

Protein	Heat-induced unfolding				
	Δ*H*_m_	*T*_m_	Δ*C*_p_	Δ*G*_D_ (kcal.mol^-1^)	
	(kcal.mol^-1^)	(K)	(kcal.mol^-1^.K^-1^)	37 ^0^C	10 ^0^C
**hGBP1**	164 ± 4	322.3 ± 0.4	5.9 ± 0.2	4.8 ± 0.4	5.2 ± 0.4
**hGBP1+GppNHp**	189 ± 5	325.2 ± 0.2	6.5 ± 0.2	6.5 ± 0.5	5.9 ± 0.4
**hGBP1+GDP**	148 ± 3	319.9 ± 0.4	7.5 ± 0.3	3.4 ± 0.2	0.5 ± 0.1
**hGBP1+GMP**	164 ± 6	322.8 ± 0.3	5.7 ± 0.2	5.0 ± 0.4	5.6 ± 0.4

Heat-induced denaturation studies were carried out with varying concentrations of urea to obtain Δ*H*_m_ versus *T_m_*. Δ*C*_p_ was evaluated from the slope of Δ*H*_m_ and *T*_m_ plot. These thermodynamic parameters were used to calculate the Δ*G*_D_ at different temperatures using Eq. 3.

The energy diagram for GDP formation from GDP-bound enzyme monomer is shown in Fig. 4, as it represents a crucial step of the overall reaction. Since the protein does not dimerize with external GDP [7], GDP-bound enzyme dimer formed as a catalytic complex after the first hydrolysis, is expected to be less stable, and has higher free energy than that of GDP-bound enzyme monomer. The activation energy associated with the forward reaction of this step will determine the amount of the product. As shown earlier, the hGBP1.GDP complex is found to be less stable at 10°C compared to at 37°C. As a result, at lower temperature the activation energy (E_4_^LT^) associated with GDP formation from hGBP1.GDP complex would be lower than that of the higher temperature (E_4_^HT^). This is consistent with higher GDP formation at low temperature. The difference in the free energy between the hGBP1.GDP complex and the product at higher temperature is much smaller than at the lower temperature. Hence, there is a strong reversibility between the hGBP1.GDP complex and the product at higher temperature. This is consistent with lower amount of GDP at higher temperature and the temperature dependent kinetic studies (-ve and zero activation energies at high and low temperature, respectively), which is described earlier. These results further support the formation of GDP shown in Fig. 6, and explain how temperature regulates the formation of GDP. Thus, the data provide an important thermodynamic insight as to why GDP became a significant product at lower temperature. Although, the biological role of the product formation in hGBP1 is not yet known, it is possible that higher GDP at low temperature could be an indication of involvement of GDP in defense mechanism under stress conditions. For example, viral infections such as H1N1, cold, flu, sendai etc. [16], [17] and Wilson’s Temperature syndrome are known to alter body’s temperature and hypothermia is also seen [25], [26]. However, further studies are required to broadly assess the role of GDP and GMP formation in hGBP1 under various conditions, such as changes in pH, UV-radiation, microbial infections etc.

### Stability of the Protein in the Presence of the Substrate Analogue and Biochemical Assays

To investigate whether the stability of the protein upon binding with the substrate is related to the product formation, we carried out steady-state assays of the wild type and mutant proteins using radiolabeled (α-^32^P) GTP. It is observed that the deletion of the C-terminal helical domain in hGBP1 decreases the *T*_m_ considerably (unpublished data) indicating that the interaction between the two domains are important for the stability of the protein. In the x-ray crystal structure of the full-length hGBP1 with GppNHp, Ser157 (globular domain) and Glu313 (helical domain) are found to interact at a distance of ≤3.5 Å and have been mutated to Ala. These mutants were also used to check whether the activity can be correlated to the stability of the proteins upon binding with the substrate. In these experiments, the substrate was used in excess over the enzyme so that multiple turnovers can be examined. The formation of GDP and GMP were found to increase with increasing concentrations of GTP. The steady-state kinetic parameters for the mutant proteins are shown in Table 3. Like wild type protein, the data for GDP in the mutants were fitted to a Hill equation with n (Hill coefficient) ∼1.1 (Fig. S1) indicating that the first phosphate cleavage is not associated with the stimulation of the activity. The value of n with greater than one for GMP formation in the mutant proteins indicates that the stimulation of the activity occurs during the cleavage of the second phosphate, and dimeric proteins are essential for GMP formation, similar to that observed for wild type protein [12]. These data clearly imply that the mechanism of GTP hydrolysis in the mutant proteins did not alter compared to wild type. The catalytic efficiency (*k*_cat_/*K*_m_) for GDP formation in Glu313Ala is decreased by ∼2 fold compared to wild type, but for GMP it is increased by ∼1.5 fold. The decrease and increase in the catalytic efficiency for GDP and GMP respectively for Glu313Ala compared to wild type is consistent with Fig. 6, where after the first hydrolysis there is a competition between the dissociation of GDP-bound enzyme dimer and second hydrolysis. The decrease in the catalytic efficiency for GDP in Glu313Ala compared to wild type suggests that Glu313 plays a role in the dissociation of GDP-bound enzyme dimer and thus increases GDP. But for Ser157Ala, the catalytic efficiency for GDP is increased by ∼1.5 fold compared to wild type suggesting that Ser157 is important in preventing the dissociation of GDP-bound enzyme dimer. The catalytic efficiency for GDP and GMP was reported in the case of hGBP1^311^, a truncated protein of hGBP1 (where the helical domain has been deleted) and D108A, a catalytically important mutant (which reduces GMP formation compared to wild type) [12]. It was observed that the catalytic efficiency for GDP was increased by almost 1.5–2 fold compared to wild type, whereas for GMP it was decreased by about 2 fold, which is similar to Ser157Ala. The change in the catalytic efficiency in the mutant proteins may be due to the alteration in the assembly of dimeric protein upon binding with the substrate. All these biochemical data are consistent with the mechanism proposed in Fig. 6.

**Table 3:** Steady-state kinetic parameters of the wild type and mutant hGBP1 proteins.

Protein	*k*_cat_^GDP^/*K*_m_^GDP^(µM^−1^. min^−1^)	n^GDP^	*k*_cat_^GMP^/*K*_m_^GMP^ (µM^−1^. min^−1^)	n^GMP^
whGBP1^a^	0.0175	1.1±0.18	0.13	1.4±0.1
Glu313Ala	0.008	1.1±0.19	0.21	1.9±0.3
Ser157Ala	0.03	1.2±0.16	0.08	1.4±0.2

The experiments were carried out by incubating the enzyme with a fixed amount of radiolabeled (α-^32^P) GTP and varying concentrations of unlabelled GTP as described in the experimental procedures. The apparent *K_m_, k_cat_* and n (Hill co-efficient) for both GDP and GMP formation were analyzed using a Hill equation, Rate  = *k_cat_* [E_0_].[GTP]^n^/(*K_m_* ^n^ + [GTP]^n^). The quality of fit was judged by a theoretical line drawn through the experimental data points and highest confidence limit.

a reported [12].

**Figure 9:**
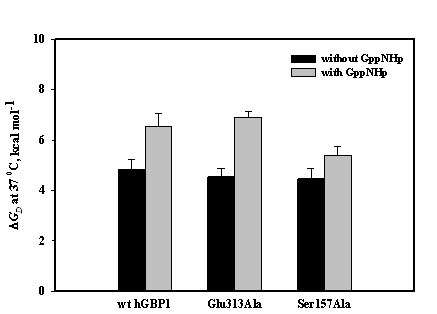
▵*G*_D_ of wild type and mutant hGBP1 in the absence and presence of GppNHp. Heat-induced denaturation studies of wild type and mutant proteins were carried out with or without GppNHp. The experimental data were fitted using Eq. 2 to obtain ▵*H*_m_ and *T*_m_. ▵*C*_p_ of the mutant proteins was assumed to be same as in wild type. With these values, the ▵*G*_D_ at 37 °C was calculated using Eq. 3.

To understand whether the above difference in the product formation can be correlated to the stability, we carried out heat-induced unfolding studies of the wild type and mutant proteins separately with or without GppNHp. As already known, wild type hGBP1 exists as a dimer in the presence of GppNHp and dimerisation has been found to be essential for GMP formation and stimulation of the activity [13]. The heat-induced unfolding studies of wild type protein without denaturant were described in the earlier section and the data were analyzed to obtain the thermodynamic parameters Δ*H*_m_ and *T*_m._ Like wild type, these mutants exist as dimer in the presence of the analogue, as observed from the analytical gel filtration analysis (Table S1). To determine the Δ*G*_D_ of the mutant proteins, similar heat-induced unfolding studies were carried out with and without GppNHp and the thermodynamic parameters were determined (Fig. 9). Analysis shows that like wild type the binding of the analogue increases the stability of the mutant proteins. This is perhaps essential for a substrate induced conformational switch that leads to dimerization of the protein. The increase in the stability may be contributed mainly due to tight packing of the nucleotide induced dimeric protein. A careful examination reveals that the relative difference in the Δ*G*_D_ between the analogue free and bound proteins for Ser157Ala is lower than wild type (1.7 kcal/mol vs 0.9 kcal/mol for wt vs Ser15Ala respectively) but for Glu313Ala it is higher than wild type (2.4 kcal versus 1.7 kcal/mol for Glu313Ala versus wt respectively). This suggests that the difference in Δ*G*_D_ between the analogue free and bound proteins plays an important role in the product formation; larger difference (higher stability) favours for the second hydrolysis i.e. GMP formation, but smaller (lower stability) favours for the dissociation of GDP-bound enzyme dimer resulting more GDP. This is in agreement with the steady-state kinetic assays, where Glu313Ala shows higher catalytic efficiency for GMP (consequently lower catalytic efficiency for GDP) compared to wild type but Ser157Ala shows lower for GMP (consequently higher catalytic efficiency for GDP). This is consistent with earlier hypothesis, where after the first hydrolysis there is a competition between the further hydrolysis of GDP-bound enzyme dimer to GMP and the dissociation of this dimer into monomer leading to GDP formation.

### Conclusions

In the present study, we have examined the effect of temperature on the GTPase activity of a complex enzyme hGBP1 that has unique feature of hydrolyzing GTP to a mixture of GDP and GMP, with GDP being significant at 15°C. The temperature dependent kinetic data reveal that GDP formation occurs through the reversible dissociation of GDP-bound enzyme dimer to GDP-bound enzyme monomer, which further reversibly dissociates into the free enzyme and product. This is supported by the GTPase assays in the presence of external GDP. Urea-induced unfolding studies of different catalytic complexes show that unlike all complexes the stability of the GDP-bound protein decreases significantly at low temperature and plays a crucial role in the product formation. The decrease in the stability of GDP-bound protein after the first hydrolysis at low temperature (10°C) compared to at high (37°C) illustrates why GDP becomes significant at low temperature. Heat-induced unfolding studies on the wild type and mutant proteins suggests that a relatively stable dimer with respect to its monomer favours GMP formation, which may be required for the stimulation of the activity. This finding is consistent with the biochemical analysis of Glu313Ala and Ser157Ala, where the catalytic efficiency for GMP is higher and lower respectively than wild type. Thus, the study provides the first thermodynamic insight as to how the stability of an intermediate catalytic complex regulates the product formation in hGBP1.

## Supporting Information

Figure S1 Steady-state kinetic assays for Glu313Ala and Ser157Ala. The experiments were carried out by incubating the enzyme with a fixed amount of radiolabeled [α-^32^P] GTP and varying concentrations of unlabelled GTP. The data were fitted using a Hill equation, Rate  = *k*_cat_ [E_0_].[GTP]^n^/(*K*_m_^n^ + [GTP]^n^) to obtain the apparent *K*_m_, *k*_cat_ and n (Hill co-efficient). The quality of fit was judged by a theoretical line drawn through the experimental data points and highest confidence limit.
(TIF)

Table S1 Oligomerization of wild type and mutant proteins in the absence and presence of GTP-analogue (GppNHp) based on the analytical size exclusion chromatography. The experiments were carried out in triplicate and the results were found to be consistent.
(DOC)
