# Supplementary figures and images for: Insight into Temperature Dependence of GTPase Activity in Human Guanylate Binding Protein-1
Source: PLoS One. 2012 Jul 11;7(7):e40487. doi: 10.1371/journal.pone.0040487 (PMC3394710; doi:10.1371/journal.pone.0040487)

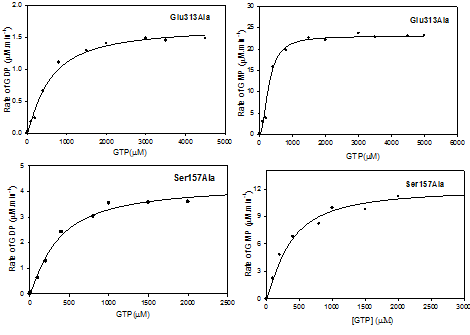

Supplement: Figure S1 — Steady-state kinetic assays for Glu313Ala and Ser157Ala. The experiments were carried out by incubating the enzyme with a fixed amount of radiolabeled [α-32P] GTP and varying concentrations of unlabelled GTP. The data were fitted using a Hill equation, Rate = k cat [E0].[GTP]n/(K m n + [GTP]n) to obtain the apparent K m, k cat and n (Hill co-efficient). The quality of fit was judged by a theoretical line drawn through the experimental data points and highest confidence limit. (TIF) [file pone.0040487.s001.tif]
