# Supplementary material for: Insight into Temperature Dependence of GTPase Activity in Human Guanylate Binding Protein-1
Source: PLoS One. 2012 Jul 11;7(7):e40487. doi: 10.1371/journal.pone.0040487 (PMC3394710; doi:10.1371/journal.pone.0040487)
Supplement: Table S1 — Oligomerization of wild type and mutant proteins in the absence and presence of GTP-analogue (GppNHp) based on the analytical size exclusion chromatography. The experiments were carried out in triplicate and the results were found to be consistent. (DOC) [file pone.0040487.s002.doc]

**TABLE S1.**

| **Protein** | **Molecular mass in the absence of analogue** | **Molecular mass in the presence of GppNHp** |
| --- | --- | --- |
| whGBP1 | ~78,000(monomer) | ~150,000(dimer) |
| Ser157Ala | ~79,500(monomer) | ~148,500(dimer) |
| Glu313Ala | ~82,000(monomer) | ~130,000(dimer) |
